# Midwifery

**Published:** 1836-07

**Authors:** 


### MIDWIFERY.

*Case of Cæsarean Operation performed three times successfully on the same Individual.* By Dr. Michaelis, of Kiel.

The patient was born in 1795, and during her childhood suffered from rickets to such a degree that she could scarcely walk alone till twelve years of age. The lower extremities, the spine, and pelvis, were much distorted. Her height is four feet three inches;* the trunk is disproportionately long. On examination per vaginam, the antero-posterior diameter, from the lower edge of the symphisis pubis to the promontory, is about two and a half inches; from the upper edge of the symphisis pubis to the promontory, it is about two inches; the sacrum is nearly straight. Her first pregnancy was perfectly natural; the first pains having made their appearance, at the end of forty weeks, on the 16th of June, 1826. The os uteri dilated, and the membranes ruptured during the night. The Cæsarean operation was determined on the evening of the following day by the two medical men who had been summoned, but, as the necessary preparations could not be made immediately, it was deferred till the morning of the 18th; twelve leeches were applied to the lower part of the abdomen the night before, and some emulsion with nitre given to allay a feverish attack.

*First Operation.* On the morning of the 18th June, she was placed on a table nearly horizontally, on account of the abdomen being very pendulous. Dr. Seidel applied a napkin dipped in oil around the spot selected for the incision, and pressed the uterus, which was much inclined to one side, into the median line of the body, where he held it firm. The incision commenced in the linea alba, two inches below the umbilicus, and terminated two inches above the symphysis pubis. The divided integuments instantly separated a hand's breadth from each other. As the tension of the abdominal parietes was still so considerable as to prevent the finger being introduced, the peritoneum was divided by passing a director beneath, and cutting it with a blunt-ended bistoury. As soon as this was effected, Dr. Seidel pressed back some convolutions of intestine, which had protruded on both sides, so quickly that they produced no interruption to the operation.

When the uterine parietes were divided, the external wound required further dilatation before the child could be extracted. As the uterus contracted strongly, the placenta was immediately separated and brought away. A considerable hemorrhage from the edges of the uterus followed: cold water was poured upon it from a height; firm contraction followed, and the hemorrhage ceased. The child had been evidently dead some time. The lips of the wound came into such exact apposition that the suture was not deemed necessary, and they were merely held together with long strips of adhesive plaster; charpie and a common bandage completed the rest of the dressing, and the patient, who had been perfectly quiet during the whole operation, was placed in bed upon her back. A little cool drink was given her; a mixture of Decoct. altheæ, Potass. nitras, and Ol. olivæ, was ordered; and at night she took a grain of acetate of morphium. Pulse 120, small and hard. The night passed tranquilly; she slept well, slight perspiration, much thirst.

19th June. Feverish during the night. Urine passed without the catheter. The bowels not being open, she took every hour a tablespoonful of the following mixture:—℞. Decoct. Altheæ, ℥vj.; Ol. Amygd. dulc., Syrup. Rhæi, aa ℥j. M.

21st June. Bowels have been moved seven times; pain in abdomen diminished; no fever.—Half a grain of morphium was given several times daily until the 29th: she took in all one scruple.

23d June. The dressings were undone: the wound had united to the extent of three inches. The edges of the lower portion were not united, and were again brought together by fresh strips of adhesive plaster.

25th June. Felt very well: appetite good. Took Decoct. cinchonæ with camphor, and had some broth.

The only inconvenience of which the patient complained, and which continued to annoy her to the end of the month, was a scalding in passing water, which frequently produced retention of urine, requiring the catheter. An enema was necessary almost daily, to procure the necessary relief of the bowels. The lochia were regularly secreted; the milk was sparing.

In the course of three weeks the wound was completely closed and cicatrized. On the 4th of July she sat up for the first time, and on the 10th she left her bed. The cicatrix was of about the breadth of a finger; the abdomen was rather pendu-

* These numbers have been reduced to the English measure.

lous, and required a bandage. The catamenia returned in eight weeks after the operation.

Dr. Zwanck, the operator, attributed much of his success, and the remarkable absence of unfavorable symptoms, to the dressing having been made without suture, and to the free use of morphium.

*Second Pregnancy.* She menstruated for the last time on the 13th April, 1829. She quickened towards the end of August. She enjoyed good health during her pregnancy, except that the abdomen, being pendulous, hindered her somewhat in walking.

She was admitted into the Lying-in Hospital at Kiel, in December, 1829. External examination showed a strongly-marked pendulous belly: this was evidently produced by actual dilatation of the uterus, and great distention of the abdominal parietes in the immediate vicinity. The cicatrix had now extended to the length of nine inches; it was four inches broad. The fundus uteri was high above the umbilicus; the distance of the umbilicus from the pubis was full fourteen inches, (fifteen English;) whereas, in her first confinement, it was stated to be eight and a half, (nine English.) The uterus appeared to be in close contact with the abdominal parietes, and at the lower part of the cicatrix was evidently united with them for the space of an inch and a half.

In the beginning of January, 1830, she frequently complained of great distention of the abdominal integuments, at the lower part; the veins in the cicatrix were much dilated; and the little scars, where the leeches had bitten in her last pregnancy, now partially opened, and once during the night bled considerably.

*Second Operation.* Pains commenced between the 20th and 21st of January. On the morning of the 21st, the os uteri was scarcely at all dilated; an extremity could be felt through the vagina. By half-past four P.M., the os uteri was dilated about three fingers' breadth; the pains were strong, and membranes tense. As an extremity presented, and would necessarily prolapse in all probability with the cord, as soon as the membranes ruptured, it became no longer safe to delay the operation. Having therefore evacuated the bladder and rectum, it was commenced by the celebrated Wiedemann, of Kiel, in the presence of Professors Lüder and Deckmann, Dr. Michaelis, and several students. The two latter gentlemen supported the abdominal integuments with their hands. The incision, six inches in length, was made on the left side in the cicatrix, about an inch from the median line, where the child was felt most distinctly, commencing at about five inches below the umbilicus, and terminating about three inches from the os pubis. The uterus was then divided layer by layer; a few vessels spouted for a moment. The placenta appeared in the wound, which was dilated full four inches. During a pain the placenta protruded, and occupied the opening. To separate the placenta on the right side, rupture the membranes, and extract the child (which had presented with a thigh) as far as the head, was the work of a few seconds. A powerful contraction of the uterus now retained the head for a few moments, and the child's life appeared in considerable danger: gentle traction soon released it, and the placenta followed. The operation lasted barely five minutes. The child, a girl, soon cried briskly, and the uterus contracted well.

The wound of the integuments was secured by three sutures, made after Gräfe's plan, and supported with strips of adhesive plaster, and a small seton placed in the lower end. The patient, who had borne the whole operation without a murmur, complained of great pain in making the suture, and was very impatient.

After the operation the pulse was very slow and hard; her appearance was unchanged. On account of severe pains in the abdomen, (apparently after-pains,) twenty drops of Sp. Ætheris comp. and eighteen of Træ. Opii were given. She continually demanded morphium, which was not given. At eleven in the evening, as the pains were unabated, she had one grain of opium; at three-quarters past twelve she fell asleep, but was very restless from the after-pains.

Considering all circumstances, her recovery was favorable, so that, on the 21st of February, she was up for some time, and felt quite well. By the beginning of March the wound was entirely cicatrised, except a place or two in the skin. On careful examination, a fistulous opening was discovered, from which a little mucus could be squeezed. A probe which was introduced caused repeated bleeding, and at length penetrated downwards more than an inch into the uterus, which had become firmly united with the abdominal integuments. Injections into the fistula passed away immediately by the vagina, and a small quantity of muco-purulent fluid was constantly discharged from this passage. This fistulous canal resisted all attempts to make it heal until the patient left the hospital.

The union of the uterus with the abdominal integuments had so much increased this time, that, when the organ was fully contracted, it occupied a space of at least an inch and a half, and consisted therefore of its entire anterior surface. The child died on the 19th of February, of induration of the cellular tissue.

*Third Pregnancy.* The catamenia appeared in the course of a few weeks after her return, and the fistulous opening healed shortly afterwards of itself. In the middle of June, 1831, the catamenia appeared for the last time; and, at the beginning of March, 1832, she was again admitted into the Lying-in Hospital of Kiel. Her pregnancy had passed over without any peculiar inconvenience.

Early on the 28th of March pains made their appearance; by ten the same evening, the os uteri was transversely dilated to the extent of three fingers' breadth, and the membranes were very tense during a pain. The head could be distinctly felt above the pubis. The patient looked forward with confidence to the operation, but forbade most expressly the suture being made this time with Gräfe's large needle.

*Third Operation.* Having evacuated the bladder and rectum, Dr. Michaelis commenced the operation in the presence of Professors Weidemann and Deckmann, &c.; the latter supported the abdominal parietes. The spot selected for the incision was to the left of the second cicatrix, where the child could be felt most distinctly. It was made about five inches long, in an oblique direction from above and outwards, downwards and inwards. On enlarging the incision into the uterus with a blunt-pointed bistoury, the liquor amnii escaped. An elbow presented, close behind which was the left knee: this latter was brought out; as, however, the nates did not follow gentle traction, the opening was dilated somewhat, and the child was extracted without difficulty. It was a boy, and cried immediately. The placenta was quickly separated, and brought away. Till this moment no fluid had escaped into the abdominal cavity, but now, as the uterus contracted imperfectly, a stream of blood issued from the wound: this was soon stopped by pouring water from a considerable height above it. No convolution of intestine protruded. The pulse was slow, not small. Some wine and Tinct. Opii were given her.

The healing of the wound advanced regularly until the 16th of May: there were some little places which had not cicatrised over, but these were quite closed in a few days more. On the 25th of May, Dr. Michaelis discovered another small opening in the lower third of the wound, leading through a fistulous canal into the uterus, which was firmly united to the abdominal integuments.

27th of May, the patient left the hospital with her child in perfect health. News was received of their good health on the 10th of June, and that the fistulous opening had healed. The child died sometime afterwards of scarlet fever.

*Neue Zeitschrift für Geburtskunde*, B. iii. H. 3. 1835.

---

### *Report of Cases at the Lying-in Hospital at Dresden, during the Years* 1833 *and* 1834.

DURING 1833, 307 patients were delivered, giving birth to 314 children; amongst which were five cases of twins and one of triplets. Artificial assistance was required in thirty-seven cases: in thirty-one of these the forceps was applied; in three, extraction with the hand was used; in one case, paracentesis of a hydrocephalus; in one, perforation; and in one the Cæsarean section.

The hand was required to be introduced for the delivery of the placenta in thirteen cases: viz. in three on account of profuse hemorrhage, and in ten on account of preternatural adhesion to the internal surface of the uterus.

Of the 314 children, 152 were males, 161 females. The sex of an embryo of two months was not ascertained.

One boy and four girls were born before the twenty-eighth week; five boys and eight girls were born between this and the full period. Three boys and one girl were born in a state of asphyxia. Eleven boys and thirteen girls were born dead. Six boys and five girls died in the hospital after birth.

The longest child born alive measured 22.37 inches;* the shortest, 14.91 inches. The heaviest weighed ten, the lightest two and a half pounds.

Of the patients, 297 were discharged in good health; two were sent to the infirmary of the town; five died.

The Cæsarean section proved fatal to the mother; the child was saved. The operation lasted seven minutes; twenty minutes more were lost in producing contraction of the uterus, to stop hemorrhage. In order to prevent loss of time in applying the bandages, some strips of adhesive plaster, three inches broad, were placed under the loins before the operation, of such a length as nearly to encircle the abdomen twice. The patient died on the fifth day, of inflammation.

In 1834, 236 women gave birth to 242 children; there having been four cases of twins, and one of triplets. A case of complete prolapsus uteri and vaginæ (not frequent) was also admitted.

Artificial assistance was required in thirty-seven labours: the forceps was applied in twenty-nine cases; extraction by the hand in four; one child was turned; in two the head was perforated; and in one case embryotomy was required. The placenta was extracted in fifteen cases.

Of the 242 children, 115 were boys, 127 were girls. One boy and one girl were born before the twenty-eighth week; twelve boys and thirteen girls between this period and the full time. Four boys and one girl were born in a state of asphyxia; twelve boys and eight girls were born dead. Eight boys and two girls died in the hospital after birth. The longest child measured 21.30 inches; the shortest, 14.38. The heaviest weighed nine and a half; the lightest, two pounds.

Two hundred and thirty-three patients were discharged well; eight died.

*Neue Zeitschrift für Geburtskunde*, B. iii. H. 3. 1835.

---

### *On the Acidity of the Catamenial Blood; and on the Theory of its Origin.* By M. C. Retzius, m.d., of Stockholm.

It is universally known that the menstruous fluid does not coagulate. The cause of this has been sought for in the absence of fibrine in it, said to be ascertained by chemical analysis; a result supposed to be confirmed by the slowness with which it runs into putrefaction. Osiander and others state that it can be kept even for a whole year without becoming putrid. Now, considering the speedy putrefaction of all other kinds of blood, and assuming (as was done) that the cause of this disposition was the great quantity of azote in the blood, the conclusion was very natural that, as the fibrine contains most azote, the slow putrefaction of the menstruous blood might be owing to the want of fibrine.

These opinions and inferences are opposed by Dr. Retzius, in a memoir lately published by him in the *Eyr*. He contends that, if the menstruous blood contained no fibrine, the rest of the blood of the female ought to contain more than that of the male, that the female ought therefore to be more subject to inflammatory diseases, which is not the case, &c. He further maintains that the disposition of a substance to putrefaction does *not* depend on the greater or less quantity of azote contained in it; since *caffein*, which of all substances, next to urea, contains most azote, does not become putrid, whether dissolved in water or not, or even when exposed to heat. Moreover, by microscopical experiments, Dr. Retzius convinced himself that the menstruous blood contained the same globules, as to form, colour, number, &c., as other blood.

In the course of his experiments, Dr. R. was led to apply litmus paper to menstruous blood, and he was much surprised to find that the blue colour was very

* The inches are necessarily put in decimals, having been reduced from the French measure. The species of weight is not stated, but we presume that it is the Medizinal Gewicht, which is about the same as our Apothecaries' weight.—Rev.

quickly turned red by it; from whence he inferred its superior acidity to ordinary blood. By further examination, he discovered that this acid quality of the fluid depended on the presence of free phosphoric and lactic acids. If subsequent experiments confirm this fact, it is easy to explain the non-coagulation of the catamenial blood.

[The following is Dr. R.'s explanation of the mode of formation and operation of the acids.]

It is obviously nature's intention to produce a *remora* and congestion of blood in the uterine vessels. This physiological disposition depends partly on the great number and frequent anastomosis, in the uterus, of the arterial branches derived from the spermatic, uterine, and internal pudic arteries; partly on the thinner conformation of these arterial branches; partly on the condition of the veins in the same part,—their ramifications and want of valves; and partly on the greater relative volume of the aorta in the female. During this congestion, free phosphoric and lactic acid are formed in the substance of the uterus, and, when formed, alter the quantity of the blood in the congested vessels, by dissolving or otherwise modifying its fibrine, so as to prevent it being afterwards separated from it; just as fibrine, when dissolved in an acid out of the body, cannot be recovered in its original form.

Dr. Retzius further asserts that, after the evacuation of the acid blood accumulated in the uterine vessels by the natural process, if blood still continues to be discharged (as in menorrhagia,) it coagulates, because there has not been time for the formation of the acids in the uterine vessels; and he has found, in a woman affected with profuse menstruation, that, during the first three days, when there was no coagulation of the catamenial fluid, this was acid, and that afterwards it began to coagulate, and at the same time lost its acidity.

Dr. R. conjectures that, during the pregnant state, when fibrine is so necessary to the process going on in the uterus, probably no acid is formed in its blood; and he states that, at the first catamenial period after delivery, the blood is not nearly as acid as it becomes afterwards. He further remarks, as being in accordance with his views, that the formation of free acids takes place at different periods in other organs, and during the performance of other functions: as, for instance, in the preparation of the gastric juice, in the urine during the existence of morbid processes in the system of the vena porta, as in gout, &c.

[It is quite needless to inform our intelligent readers that there is much loose argument and pure hypothesis in the foregoing observations. In the present state of medical science, however, we are unwilling to pass over anything which may tend to direct the mind of the student and practitioner to a closer examination of the physiological processes taking place in the human body, and generally to the department of animal chemistry, from the cultivation of which medical science expects so much.]

*Eyr*, No. 3, 1835. *Christiania.*

---

*A new Diagnostic Sign of Pregnancy.* By Professor KLUGE, of Berlin.

DR. SOMMER, of Copenhagen, informs us, in the journal of which he is one of the editors, that Dr. Kluge, professor of midwifery in Berlin, considers as a *sure test* of pregnancy *a blueish tint of the vagina,* extending from the os externum to the os uteri. According to Dr. K., this discoloration commences in the fourth week of pregnancy, continues to increase to the period of delivery, and ceases with the lochia. The only condition considered as likely to vitiate the test is the existence of hemorrhoids in a very marked degree. Dr. Sommer convinced himself of the presence of this particular colour in pregnant women, under the direction of Professor Kluge.

[If the validity of this sign should be established, of which we take leave to doubt, its importance in certain cases must be admitted, when we consider the difficulty, not to say impossibility, of detecting pregnancy in its earliest period. It is at least evident from this, as from a thousand other indications, that the employment as well as the estimation of *physical signs* is daily increasing.]

*Journal for Medicin og Chirurgie. November,* 1835.

---

### *Use of Ergot in inducing Abortion.* By Dr. WEIHE.

A WOMAN, aged thirty-six, pregnant with her sixth child, in the fourth month of her pregnancy was attacked with uterine hemorrhage. After this had continued most violently for three days, without any appearance of expulsion of the fœtus, and when the woman was apparently dying from loss of blood, Dr. Weihe, with the view of inducing more speedy miscarriage, ordered eight grains of ergot of rye to be given every half-hour. After the third powder was taken, labour-pains came on, and she had scarcely taken the fourth before the fœtus was expelled. The patient fainted, but soon recovered: the hemorrhage ceased immediately, and she gradually recovered, although she remained exceedingly weak for a long time.

*Bibliothek for Læger.* No. 2, *(April,)* 1836.

